# The intra-nucleus integration of mitochondrial DNA (mtDNA)in cervical mucosa cells and its relation with c-myc expression

**DOI:** 10.1186/1756-9966-27-36

**Published:** 2008-09-09

**Authors:** Daozhen Chen, Wenqun Xue, Jinying Xiang

**Affiliations:** 1 Center Laboratory, Wuxi Hospital for Matemaland Child Health Care, Affiliated Medical University of Nanjing, Wuxi, 214002, PR China

## Abstract

**Objective:**

To explore the relationship between the integration of mitochondrial DNA(mtDNA) in the nuclei of cervical epithelium cells and the expression of c-myc.

**Methods:**

The expression of c-myc protein was measured by immunohistochemical test in 40 cases of the uterine cervix cancer, 30 cases of cervical intraepithelial neoplasia (CIN) and 30 cases of normal cervical epithelium; the sequence of mtDNA in the nuclei was detected by in situ hybridization technique.

**Results:**

The detection rates of mtDNA in the nuclei of cervical epithelium cells were 27.5%, 13.3% and 0% in cervical carcinoma, CIN, and normal cervical epithelium respectively. The expression rate of c-myc in cervical mucoma cells was 67% in the mtDNA sequence positive group and was significantly higher than that in the negative group (36%).

**Conclusion:**

The integration of mtDNA into the nuclei of cervical epithelium cells may be involved in the carcinogenesis of cervical epithelium cells and the expression of c-myc might be related to the integration of mtDNA sequence into nuclei of cervical epithelium cells.

## Background

**I**n recent years, several studies have found that point mutation of some tumors was relevant to that of mtDNA, but it is unclear for causal relation, which could not rule out the possibility of mtDNA integration to the nuclear genome and inducing carcinogenesis. Actually there were objective conditions for the intranuclear transfusion and integration of mtDNA and its fragments. Physical, chemical and certain biological factors may cause mtDNA mutations, the collapse of mitochondrial membrane, and give rise to mtDNA and its fragments dissociation into the cytoplasm. When the free mtDNA and its fragments in the cytoplasm generate excessivelly and the activity of DNAase DNAase-like materials is degraded, the free mtDNA or its fragments probably has the similar effect of tumorgenic virus, passing through nucleopore and randomly integrating into genome DNA. The roles of mtDNA intranuclear integration could be as follows: (1) The integration fragments or integration sites do not influence the normal function of genome and have little impact on the biological characteristics of the host cells; (2) activation of a "healthy gene" enhances the body's disease resistance and promotes biological evolution; (3) oncogene activation or anti-oncogene inhibition causes cell proliferation and differentiation out of control, which finally leads to cancerization; (4) apoptosis gene activation or anti-apoptosis gene inhibition induces cells apoptosis rapidly. More and more data indicated that mtDNA integration existed in the nuclear genome of tumor cells. Liang etc. [1] has also found the phenomenon of mtDNA fragments intranuclear integration in early glioma cells by fluorescent in situ hybridization of chromosomes. Kamimura etc. [2] detected a section of mtDNA sequence homology in the nDNA of tumor cells, which is composted of three unconsecutive sections of mtDNA: 12S rRNA, cytochrome oxidase I (COX-I) and a part of ND4L/ND4 DNA. Later Shay [3] has got the similar findings in the nuclear genome research on Hela TG cervical cancer cells. mtDNA intranuclear integration may lead to the instability of chromosome DNA and oncogene activation and/or anti-oncogene deactivation, which lead to abnormal cell proliferation and differentiation and finally result in cancerization.

Carcinoma of the uterine cervix is the second commonest malignancy in women only next to breast cancer. Activation of oncogene and inactivation of anti-oncogene are molecular basis of cancerization of cells. Some scholars [4] suggested that mtDNA, the unique genetic materal outside of chromosome, may be randomly integrated into genome DNA and activate oncogene or inactivate anti-oncogene, and finally induce the development of tumor. Previous study of our lab [5] has found that higher frequency of mtDNA mutation existed in cervical cancer. The purpose of this study was to enrich the study of molecular mechanism of cervical cancer by detecting intra-nucleus integration of mtDNA segment in cervical mucosa cells and exploring its correlation with c-myc (an important oncogene).

## Materials and methods

### Cases

40 patients with cervical cancer were collected from 2000 to 2004 for biopsy samples, including 34 cases of squamous cell carcinoma and 6 cases of adenocarcinoma. According to FIGO clinical staging standard, 13 cases were in stage I and 27 cases were in stage II; These cases were classified as histological grading standard: 9 cases in grade I, 21 cases in grade II and 11 cases in grade III. radical hysterectomy plus pelvic curettage of lymph node was performed for all these patients whose age ranged from 36 to 71 years old, and median age was 59.5 years old. 30 cases of CIN and 30 cases of normal cervical epithelia were taken as control. Patients were informed of the nature, goals, potential benefits, and risks of participating in the study and signed a written consent form approved by the Institutional Ethics Committee.

### Pathological section and staining

Tissues of cervical cancer were taken, fixed with10% formaldehyde and embedded with paraffin, HE staining or IHC were used for sections. Some tissues were taken for 5 μm frozen sections, fixed with 4% paraformaldehyde for hybridization in-situ.

### DNA hybridization in-situ

mtDNA probe sequence refered to the relative literature [6]. Probe marks adopted Roche random primer digoxin marks and reagent kits, according to the manufacture's instruction. 2 μg of restriction endonuclease HaeIII (GG ↓ CC) and HpaII (C ↓ CGG) were respectively added to 1 μg of mtDNA probe in 37°C water bath for 2 h for enzymatic digestion which was prepared for hybridization.) Dilution of the probe was 1.5 ng/μl. The hybridization solution contained: 50% deionized formamide, 0.1%N Lauroylsarcosine,0.02% SDS, 2% blocking reagent and 5 × SSC. Hybridization solution without probe was used as negative control. The steps of the hybridization in situ were as follows: (1) each section was initially treated with 0.01 mol/l HCL and proteinase K at 37°C for 30 min, then washed with 0.1 mol/L glycine for 5 min and fixed with 4% paraformaldehyde for 5 min; (2) Prehybridization: 30 μl prehybridization solution was added to each section, at 37°C for 30 min; (3) Hybridization: 30 μl hybridization solution with probe was added to each section at 37°C for 16 h; (4) 30 μl digoxin antibody labeled with alkaline phosphoric enzyme was added at 42°C for 0.5 h; (5) coloration: the section was colored by NBT/BCIP for 30~60 min for microscopic examination and photograph. If the nucleus had hyacinthine staining but the intercellular substance and control hadn't such staining, it was regarded as positive expression.

## Detection of the c-myc expression by immunohistochemical test

Immunohistochemical reagent c-myc monoclonal antibody produced by U.S. Symed (purchased from Beijing Zhongshan Biological Technology Company, China). Operating steps: (1) the section was dewaxed and then put into water. (2) repaired for 20 min by hot platform. (3) The normal horse serum was added to section for 20 min at 1:50 dulitions for blocking) (4) the section was incubated with monoclonal antibody at 1:60 dulitions at 4°C overnight; (5) 1:120 double (secondary) antibody was added at room temperature for 60 min (6) SAHRP 1:150 for 60 min at room temperature. (7) DAB staining. (8) Hematein double staining, dehydration, transparent mount. The standard of c-myc positive reaction: the reaction product of c-myc positive reaction was brown particles, distributed in cell nucleus, it was regarded as positive expression if the percentage of possive cells was more than 30% under 10 high power fields.

### Statistical methods

The statistical analysis was performed by using SAS (6.12_version)) statistical software. The statistical comparisons between groups were performed by χ^2 ^test, *P *< 0.05 was considered statistically significant.

## Results

### Intranucleus integration of mtDNA sequence

**Figure 1:**
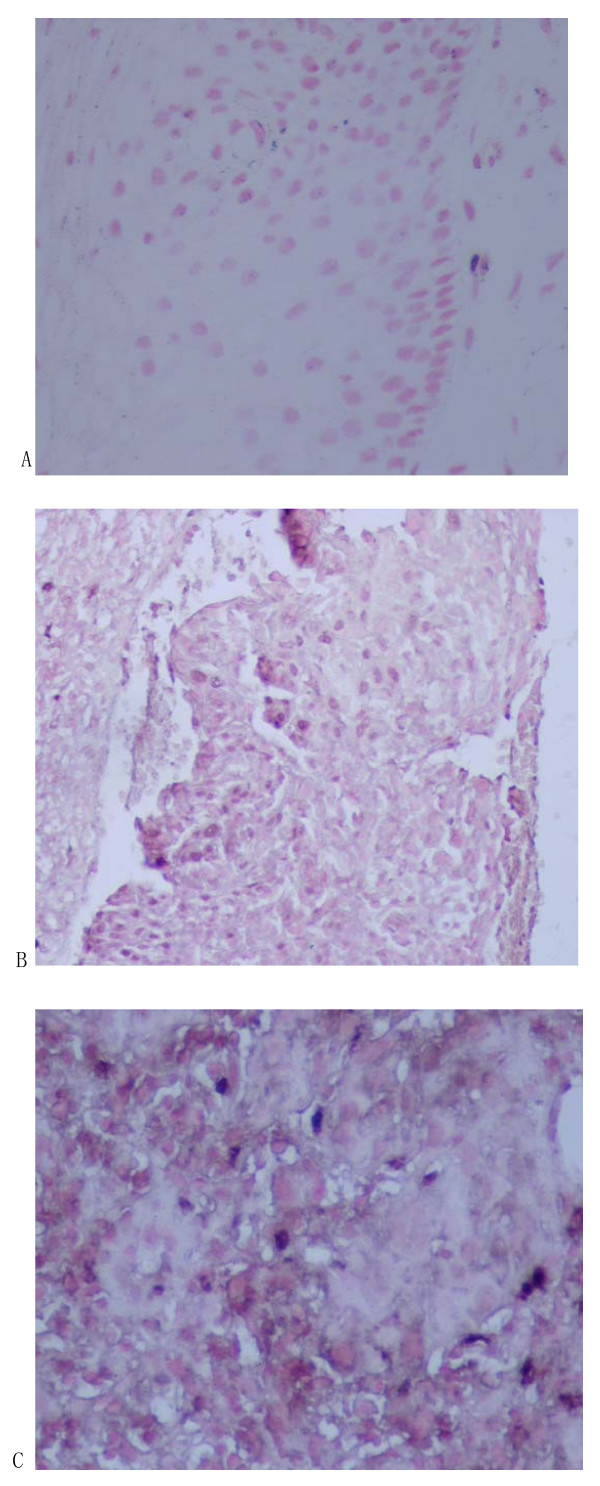
Integration of mtDNA in the nuclei of cervical epithelium cells detected by in situ hybridization The nucleus had hyacinthine staining but the intercellular substance and control hadn't such staining, it was regarded as positive expression. (A: normal cervix tissure; B: CIN tissure; C: cervical cancer tissure)

mtDNA sequence was detected in 15 cases of cervical mucosa nucleus by hybridization in-situ (see Fig 1). Integration rates in normal cervix, CIN and cervical cancer were 0%, 13.3% and 27.5% respectively. Difference among the three groups was significant (χ^2 ^= 9.054, *P *< 0.05, see Table 1).

**Table 1:** Integration of mtDNA in nuclei of cervical epithelium cells

Groups	n	+	-	integration rate (%)	p value
Cervical carcinoma	40	17	23	42.50%	χ^2 ^= 9.054(P < 0.05)
CIN	30	4	26	13.30%	χ^2 ^= 9.054(P < 0.05)
Normal cervix	30	0	30	0%	

### C-myc oncogene expression

**Figure 2:**
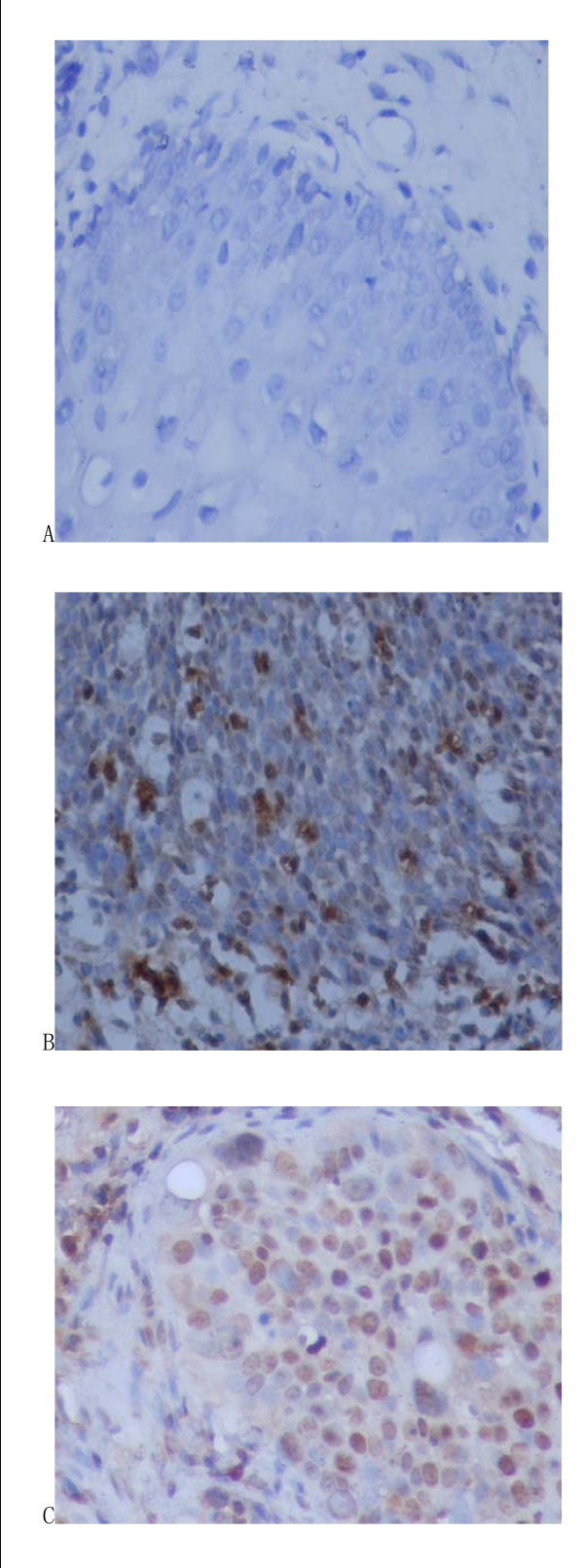
**The expression of c-myc in cervical epithelium cells detected by immunohistochemical test**. The standard of c-myc positive reaction: the reaction product of c-myc positive reaction was brown particles, distributed in cell nucleus, it was regarded as positive expression if the percentage of possive cells was more than 30% under 10 high power fields. (A: normal cervix tissure; B: CIN tissure; C: cervical cancer tissure).

Positive expression was diffused in cervical squamous cell cancer but mesenchymal was negative (see Fig 2). In CIN, positive expression was seen in atypical proliferation of epithelium whereas normal epithelia and mesenchyma were negative. The expression rates of C-myc in normal cervix, CIN and cervical cancer were 7%, 33% and 73% respectively. Difference among the three groups was significant (χ^2 ^= 10.658, χ^2 ^= 27.503, *P *< 0.05, Table 2).

**Table 2:** The expression of c-myc in cervical epithelium cells

Groups	n	positive	negative	positive rate (%)	p value
Cervical carcinoma	40	29	11	73%	χ^2 ^= 10.658(P < 0.05) χ^2 ^= 27.503(P < 0.05)
CIN	30	10	20	33%	χ^2 ^= 10.658(P < 0.05)
Normal cervix	30	2	28	7%	χ^2 ^= 27.503(P < 0.05)

### Relationship between intranuleus integration of mtDNA sequence and C-myc oncogene expression

In 100 samples, 41 were c-myc positive, of which 10 cases were positive of mtDNA hybridization in-situ nucleus staining; 59 cases were c-myc negative, of which 5 cases were positive of mtDNA hybridization in-situ nucleus staining. Difference was significant (χ^2 ^= 4.81, *P *< 0.05) (Table 3)

**Table 3:** Relationship between integration of mtDNA in nucleus and the expression of c-myc gene

	Sequence intranuleus integration (n)		
		
c-myc oncogene expression (n)			p value
	negative	positive	
negative	54	5	χ^2 ^= 4.81(P < 0.05)
positive	31	10	χ^2 ^= 4.81(P < 0.05)

## Discussion

Proper insertion of mitochondria gene into nucleus genome is important in biological evolution, but improper insertion may be one of the main causes of certain genetic diseases, malformation or tumors. Hu Yide et al [7] adopted gene transfer technology to transfect mtDNA fragment to mouse NIH3T3, which induced cells malignant transformation. It suggested that mtDNA integration in nucleus was an important factor to promote cells cancerization. Ling Xianlong et al [8] also found intranucleus integration of mtDNA fragment in cell nucleus of gastric cancer. Shay et al [9] found CoIII of mtDNA in Hela TG cells arranged in c-myc gene. The resulting mRNA contained not only genetic information from c-myc, but also from CoIII. Our lab had similar findings in FISH of Hela cells of cervical cancer cultured in vitro.

C-myc oncogene was located in chromosome 8q24, total length 6~7 kb. It had 3 exon, coding protein consists of 49 amino acid residues, molecular weight reaches 64/67 kDa. C-myc over-expression and proliferation was often found in cervical cancer tissue. Ngan [10] adopted immunohistochemical technology to study 45 cases of normal cervical tissue, 38 cases of stage I CIN, 37 cases of stage II CIN and 43 cases of stage III CIN: The results showed that c-myc expression was active in poorly developed cells. It suggested that in CIN evolution, c-myc was an important proto-oncogene. Aoyama [11] tested various pathological cervical tissues with PCR. He found that c-myc oncogene was easily proliferated and (or) over-expressed. Present study first used IHC technology to explore c-myc expression in cervical tissues. Data showed that c-myc expression decreases gradually in cervical cancer, CIN and normal cervical tissue. With the increase of malignancy, positive expression became stronger, low differentiation squamous cell cancer was stronger than high differentiation one and grade III CIN was stronger than grade I CIN, which basically conformed to the reports in literature [1].

In order to exploring the correlation between c-myc oncogene expression and mtDNA integration into genome, DNA hybridization in-situ on frozen sections was also used in present study. The results showed that in the course of chronic cervical inflammation →CIN →cervical cancer, detection rate of mtDNA sequence arranged in DNA genome increased in turn, which suggested its relation with the development of cervical cancer. C-myc gene expression rates of cervical mucosa cells were 67% in mtDNA detection group and 36% in non-detection group respectively. Thus, We infered that acted by certain physical, chemical and biological factors, mtDNA mutation took place. Mutation in D-LOOP may change affinity of trans factors related to mitochondria DNA and replication, copy number of mtDNA therefore increases obviously, normal metabolic balance was damaged, free mtDNA and its fragments were excessive and activity of nucleic acid catabolic enzymes in cells decreases meantime, mtDNA and its fragments were free outside mitochondria, it had similar effect of cancerous virus, arranged randomly into nucleus genome and activated oncogene or inhibited anti-oncogene, which influenced cell proliferation and differentiation and developed tumor.

Research already found that nucleus DNA and mtDNA might wander in cells [12]. Mitochondria RNA can be reversely transcripted into mtDNA in cytoplasm, there is nucleopore on membrane and DNA ligase in nucleus. So integration of mtDNA in nucleus gene may induce canceration of cells, change of mitochondria structure and quantity increase. Advanced study was needed to confirm the hypothesis above.

Present study detected intranucleus integration of mtDNA fragment in cervical mucosa cells, and analyzed the correlation between mtDNA fragment integration and c-myc expression. In conclusion, We tentatively assumed that Integration of mtDNA into nuclei of cervical epithelium cells may be involved in the carcinogenesis of cervical epithelium cells and expression of c-myc gene might be related to integration) of mtDNA sequence into nuclei of cervical epithelium cells. It provided new clues to reveal molecular mechanism of cervical cancer.

## Competing interests

The authors declare that they have no competing interests.

## Authors' contributions

DC conceived of the study, and participated in its design and coordination. WX carried out immunohistochemical test, drafted the manuscript and collected all of cervical cancer samples. JX carried out the DNA hybridization in-situ, participated in the design of the study and performed the statistical analysis. All authors read and approved the final manuscript.
